# *Toxoplasma gondii* and *Neospora caninum* infections in South American camelids in Switzerland and assessment of serological tests for diagnosis

**DOI:** 10.1186/s13071-020-04128-9

**Published:** 2020-05-14

**Authors:** Walter Basso, Elena Sollberger, Gereon Schares, Susanne Küker, Flurin Ardüser, Gaia Moore-Jones, Patrik Zanolari

**Affiliations:** 1 Institute of Parasitology, Vetsuisse-Faculty, University of Bern, Länggassstrasse 122, 3012 Bern, Switzerland; 2 Clinic for Ruminants, Vetsuisse-Faculty, University of Bern, Bremgartenstrasse 109a, 3012 Bern, Switzerland; 3 Friedrich-Loeffler-Institut, Federal Research Institute for Animal Health, Institute of Epidemiology, Südufer 10, 17493 Greifswald-Insel Riems, Germany; 4 Veterinary Public Health Institute, Vetsuisse-Faculty, University of Bern, Schwarzenburgstrasse 161, 3097 Liebefeld, Switzerland; 5 Centre for Fish and Wildlife Health (FIWI), Vetsuisse-Faculty, University of Bern, Längassstrasse 122, 3012 Bern, Switzerland

**Keywords:** Alpaca, *Vicugna pacos*, Llama, *Lama glama*, ELISA, Immunoblot, Toxoplasmosis, Neosporosis

## Abstract

**Background:**

Little is known about the epidemiology of *Toxoplasma gondii* and *Neospora caninum* infections in alpacas (*Vicugna pacos*) and llamas (*Lama glama*) outside South America. The study aimed to estimate the seroprevalence of *T. gondii* and *N. caninum* infections in South American camelids (SAC) in Switzerland, to optimize serological tests for SAC and to identify risk factors, which may favour infection.

**Methods:**

A total of 571 sera from 132 Swiss farms (374 alpacas and 197 llamas, mean 4.3 animals/farm) were obtained. Four commercial enzyme-linked immunosorbent assays (ELISA) for detecting antibodies against *T. gondii* (ID Screen^®^ Toxoplasmosis Indirect (TOXO-MS)) or *N. caninum* (i.e. ID Screen^®^ Neospora caninum Indirect Multi-species (NCS-MS); ID Screen^®^ Neospora caninum Competition (NCC) and ID Screen^®^ Neospora caninum Indirect (NCS)) were first assessed for their use on SAC comparing their results with those in immunoblot, and optimizing cut-offs. Subsequently, two kits (TOXO-MS and NCS-MS) were selected for seroprevalence estimation. Additionally, a risk factor analysis for infection was performed on 41 farms, which agreed to participate in a web-based survey.

**Results:**

Three kits (TOXO-MS, NCS-MS and NCC) showed almost perfect agreement (kappa > 0.901) with immunoblot results when the cut-offs were optimized, and one kit (NCS) proved not to be useful for detecting *N. caninum* seropositive SAC. By TOXO-MS ELISA, 82.3% (308/374) of the alpacas and 84.8% (167/197) of the llamas were seropositive for *T. gondii*, and 131/132 (99.2%) farms had seropositive animals. By NCS-MS ELISA, 3.5% (13/374) of the alpacas and 2.5% (5/197) of the llamas evidenced antibodies against *N. caninum*, and 9.1% (12/132) of the farms had seropositive animals. The variables “age” and “female sex” were identified as risk factors for *T. gondii* infection and “absence of cats in the farm during the last two years” as a protective factor. No risk or protective factors for *N. caninum* infection could be identified.

**Conclusions:**

This nationwide cross-sectional study demonstrated for the first time the presence of antibodies against *T. gondii* and *N. caninum* in the Swiss SAC population, highlighting a high seroprevalence for *T. gondii*, the presence of cats as a risk factor and suggesting that SAC meat might represent an additional infection source for humans.
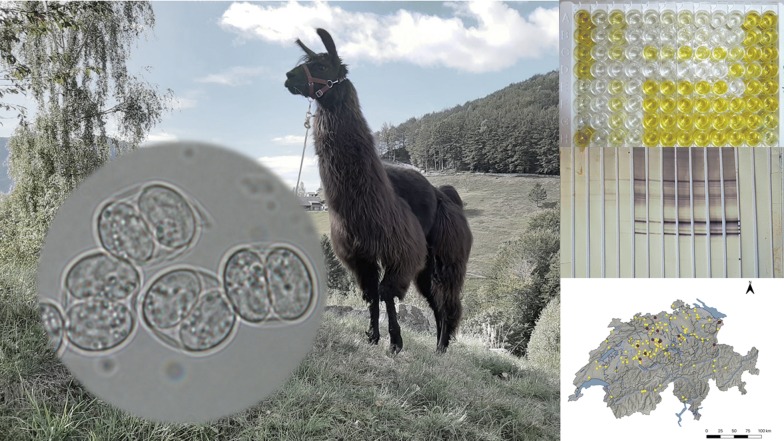

## Background

*Toxoplasma gondii* and *Neospora caninum* (Apicomplexa: Sarcocystidae) are worldwide distributed cyst-forming coccidian parasites, which represent important causes of abortion and congenital infections in ruminants [1, 2]. Both parasites have heteroxenous life-cycles, with sexual development and oocyst production in the intestine of the definitive hosts and asexual development in extraintestinal tissues of the intermediate hosts. Only felids are natural definitive hosts for *T. gondii* [1] and only domestic dogs, dingoes, coyotes and wolves have been identified as definitive hosts for *N. caninum* so far [2]. Whereas cattle and other ruminants are the main intermediate hosts of *N. caninum*, all warm-blooded species (birds and mammals, including humans) are potential intermediate hosts of *T. gondii* [1, 2]. Ruminants may become infected with these protozoan parasites either horizontally through ingestion of oocysts shed by the definitive hosts with the faeces, or vertically by transplacental parasite transmission from the dam to the foetus [1, 2]. Carnivorous and omnivorous animals can also become infected through consumption of tissues from infected hosts containing cyst stages [3].

Infections with both parasites were described in numerous domestic and wild animal species, with variable clinical manifestations. *Toxoplasma gondii* infections are frequently asymptomatic but they may be associated with fatal disease in some hosts, including humans. Certain animal species such as Pallas’ cats [4], meerkats [5], Australian marsupials [6, 7] and New World primates [8] are highly susceptible to clinical toxoplasmosis and can develop fatal generalized infections. Additionally, *T. gondii* is considered one of the most important causes of abortion in small ruminants [9, 10], but noteworthy, it has no epidemiological significance as an abortifacient agent in cattle [1]. In humans, toxoplasmosis is considered one of the most common zoonoses worldwide and can cause serious illness especially after congenital infections or in immunosuppressed patients [1, 11]. Primary *T. gondii* infections in women during pregnancy may be associated with prenatal infection and severe damage to the foetus (including hydrocephalus, intracerebral calcifications, mental retardation, chorioretinitis and death). In immunosuppressed patients, chronic infections can be reactivated leading to encephalitis or generalized toxoplasmosis [1, 11]. In recent years, *T. gondii* infections were also regarded as common cause of ocular disease in postnatal infections both in immunosuppressed and immunocompetent individuals [12]. In contrast to *T. gondii*, *N. caninum* has no zoonotic character but it is regarded as one of the major causes of abortion in cattle worldwide [2]; besides, it can also cause reproductive failure (i.e. abortion, stillbirths and perinatal mortality) in other ruminant species such as goats [13–15], sheep [16] and deer [17]. Furthermore, it represents an important cause of neuromuscular disease and death in dogs [2, 13, 17].

Little is known about the meaning of toxoplasmosis and neosporosis in South American camelids (SAC) outside South America. Only few studies worldwide showed that *T. gondii* and *N. caninum* infections may occur in SAC (Tables 1 and 2), and that these infections may be occasionally associated with abortions [18–20] as well as with fatal generalized infection [21].

**Table 1:** Seroprevalence of *T. gondii* antibodies in South American camelids

Country (Region)	SAC species	Test (commercial provider)	Cut-off	No. positive/No. examined	% Positive	Reference
Peru (Lampa, Puno)	Alpaca	IHA (Toxotest, Wiener Lab.)	1:16	89/200	44.5	[48]
	Llama			38/136	27.9	
Peru (Canchis, Cusco)	Alpaca	IFAT	1:50	97/272	35.7	[49]
Peru (Peruvian Andes, Central and South Sierra)	Llama	IFAT Conjugate: anti-llama IgG-FITC (VMRD) + confirmation of positive results by Western blot	1:50	19/43	44.2	[69]
	Vicuna			11/200	5.5	
Peru (Puno, South-Eastern Peru)	Alpaca	Immunoblot *T. gondii*		20/675	3.0	[30]
	Llama			7/81	8.6	
	Vicuna			3/114	2.6	
Peru (Central Sierra)	Alpaca	IHA (Toxotest, Wiener Lab.).	1:16	42/200	21.0	[50]
Peru (Central Sierra)	Alpaca	IFAT Conjugate: anti-llama IgG-FITC (VMRD)	1:200	22/258	8.5	[70]
Peru (Melgar, Puno)	Llama	IFAT	1:200	16/107	14.9	[71]
Peru (Central and South Peruvian Andes)	Alpaca	IFAT Conjugate: anti-llama IgG FITC (VMRD)	1:200	706/2874	24.6	[57]
	Llama			460/1845	24.9	
Argentina (Jujuy)	Llama	IFAT Conjugate: anti bovine IgG FITC (Sigma-Aldrich)	1:50	44/308	14.3	[37]
Chile	Alpaca	IHA (in-house)	1:16	32/447	7.2	[38]
Chile	Alpaca	MAT	1:25	15/127	11.8	[39]
	Llama			49/113	43.3	
USA (Northwest: Oregon, Washington State and Idaho)	Llama	MAT	1:25	95/283	33.5	[41]
USA Virginia	Alpaca	MAT	1:25	6/16	37.5	[40]
Germany (Hesse)	Alpaca	Immunoblot *T. gondii*		4/12	33.3	[30]
	Llama			10/20	50.0	
Czech Republic	Alpaca	ELISA (ID Screen Toxoplasmosis indirect multispecies ID.vet)	S/P (%) ≥ 50%^a^	1/1	100	[42]
	Llama			7/8	88.0	
	Alpaca	IFAT Conjugate: anti-llama IgG FITC (VMRD)	1:50	0/1	0	
	Llama			4/8	50.0	

^a^S/P (%) calculated according to the formula: S/P %= (OD sample/OD positive control) × 100, OD optical density

*Abbreviations*: cELISA, competitive-inhibition enzyme-linked immunosorbent assay; IFAT, indirect fluorescent antibody test; FITC, fluorescein isothiocyanate conjugate; IHA, indirect hemagglutination; MAT, modified agglutination test

**Table 2:** Seroprevalence of *N. caninum* antibodies in South American camelids

Country Region	SAC species	Test (commercial provider)	Cut-off	No. positives/No. examined	% Positives	Reference
Peru Highlands, Central and Southern Peru	Alpaca	IFAT conjugate: anti-llama IgG FITC (VMRD)	1:50	39/92	42.4	[58]
	Llama		1:50	39/212	18.4	
Peru Highlands, different regions	Alpaca	IFAT conjugate: anti-llama IgG–FITC (VMRD) + confirmation of positive results by Westernblot	1:50	14/78	17.9	[59]
	Llama			17/73	23.3	
Peru Puno, South-Eastern Peru	Alpaca	Immunoblot *N. caninum*		17/675	2.5	[30]
	Llama			1/81	1.2	
	Vicuna			0/114	0	
Peru Yauli, Central Sierra	Alpaca	IFAT conjugate: anti-llama IgG FITC (VMRD)	1:100	5/175	2.9	[72]
Peru Central and South Peruvian Andes	Alpaca	IFAT conjugate: anti-llama IgG FITC (VMRD)	1:100	425/2874	14.8	[57]
	Llama			153/1845	8.3	
Peru Arequipa, South Peruvian Andes	Vicuna	ELISA, Chekit Neospora – cELISA (IDEXX) + confirmation of positive results by Westernblot		2/207	1.0	[73]
	Alpaca			2/571	0.3	
	Llama			0/43	0	
Peru Huancavelica	Llama	IFAT conjugate: anti-llama IgG FITC (VMRD)	1:100	12/98	12.2	[56]
Peru Huancavelica	Alpaca	IFAT	1:100	47/288	16.3	[55]
Argentina Jujuy	Llama	IFAT conjugate: anti bovine IgG FITC (Sigma)	1:50	3/308	1.0	[37]
Australia South	Alpaca	ELISA, *N. caninum* Antibody Test Kit ELISA (IDEXX) + Protein G conjugate (Zymed Recombinant Protein G HRP Conjugate, Invitrogen)		0/182	0	[53]
Australia New South Wales and Victoria	Alpaca	ELISA, *N. caninum* Antibody Test Kit – cELISA (VMRD)	%I ≥ 30%^a^	3/100	3.0	[24]
Germany Hesse	Alpaca	Immunoblot *N. caninum*		0/12	0	[30]
	Llama			0/20	0	
Czech Republic	Alpaca	ELISA, *N. caninum* Antibody Test Kit – cELISA (VMRD)	%I ≥ 30%^a^	0/1	0	[42]
	Llama			1/8	12.5	
	Alpaca	IFAT conjugate: anti-llama IgG FITC (VMRD)	1:50	0/1	0	
	Llama			1/8	12.5	

cELISA: competitive-inhibition enzyme-linked immunosorbent assay, IFAT: indirect fluorescent antibody test; ^a^ %I (% inhibition) calculated according the formula: %I = 100 − (OD sample × 100/mean OD negative control), OD optical density, S/P (%) calculated according to the formula: S/P = (OD sample/OD positive control) × 100; FITC: fluorescein isothiocyanate conjugate

South American camelids are of great economic importance in the Andean Region of South America, where they are mainly bred for meat and fibre and reproductive problems may impair their breeding, causing significant economic losses [18]. Moreover, meat from infected SAC may be a potential infection source of *T. gondii* for humans, as it is often consumed undercooked. In Europe, the popularity and interest for SAC has shown a notable increase in the last years. Llamas (*Lama glama*) and alpacas (*Vicugna pacos*) are bred not only for fibre and meat production, but also for other purposes such as landscape maintenance, trekking, animal-assisted activities and therapy and also as companion animals [22, 23]. Llamas have also been used as guard animals in sheep herds against predation by lynxes in Switzerland [22] and coyotes in the USA [24] due to their natural aggressive behaviour against these animals. While at the beginning of year 2000 a total population of only 1622 SAC (623 alpacas and 999 llamas) and 257 breeders were registered in Switzerland [22], current records from the Swiss Federal Office for Statistics, revealed a total of 6739 SAC (3759 alpacas and 2980 llamas) in 2018, and a further significant increase (+1.3%) in the alpaca population was estimated for 2019 [25]. This new situation represents a challenge for many veterinary practitioners in Europe, which are not familiar with the special management and sanitary requirements of these animal species. Although SAC share some viral, bacterial and parasitic agents with domestic and wild ruminants, the clinical significance and management of the infections might be different [22, 26]. For example, while *Dicrocoelium* spp. infections are generally subclinical in cattle and small ruminants, they can cause severe liver lesions and fatal disease in SAC [26–28]. Moreover, SAC-specific nematodes (e.g. *Graphinema auchenia*, *Spiculopteragia peruviana*, *Nematodirus lamae, Lamanema chavezi* and *Camelostrongylus* spp.) are not common in Europe, and llamas and alpacas are frequently infected with nematodes from other ruminant species, but the strategies used for controlling parasites in cattle and small ruminants cannot be always directly extrapolated to SAC due to differences in pharmacokinetics, safety and efficacy for most antiparasitic drugs [26]. Therefore, a better understanding of the epidemiology and management of infectious and parasitic diseases of SAC in Europe is required.

The aims of this study were (i) to estimate the occurrence, distribution and importance of *T. gondii* and *N. caninum* infections in SAC in Switzerland, (ii) to optimize serological tests for this purpose, and (iii) to identify risk factors, which may favour *T. gondii* and *N. caninum* infections in these animal species.

## Methods

### Field samples from South American camelids and farm data

In order to estimate the nationwide seroprevalence of *T. gondii* and *N. caninum* infections in alpacas and llamas in Switzerland, a cross-sectional study including all 26 Swiss cantons was conducted. Registered SAC breeders were contacted by phone and invited to participate in the study. Each participating farm was visited, and blood samples from randomly selected animals (mean 4.3 sampled animals/farm) were obtained from the *vena jugularis.* Subsequently, serum was extracted and conserved at − 20 °C until analysis. At sampling, data on the farm (owner, register No. and location) and sampled animals (species [alpaca/llama], age and sex) were collected.

**Fig. 1:**
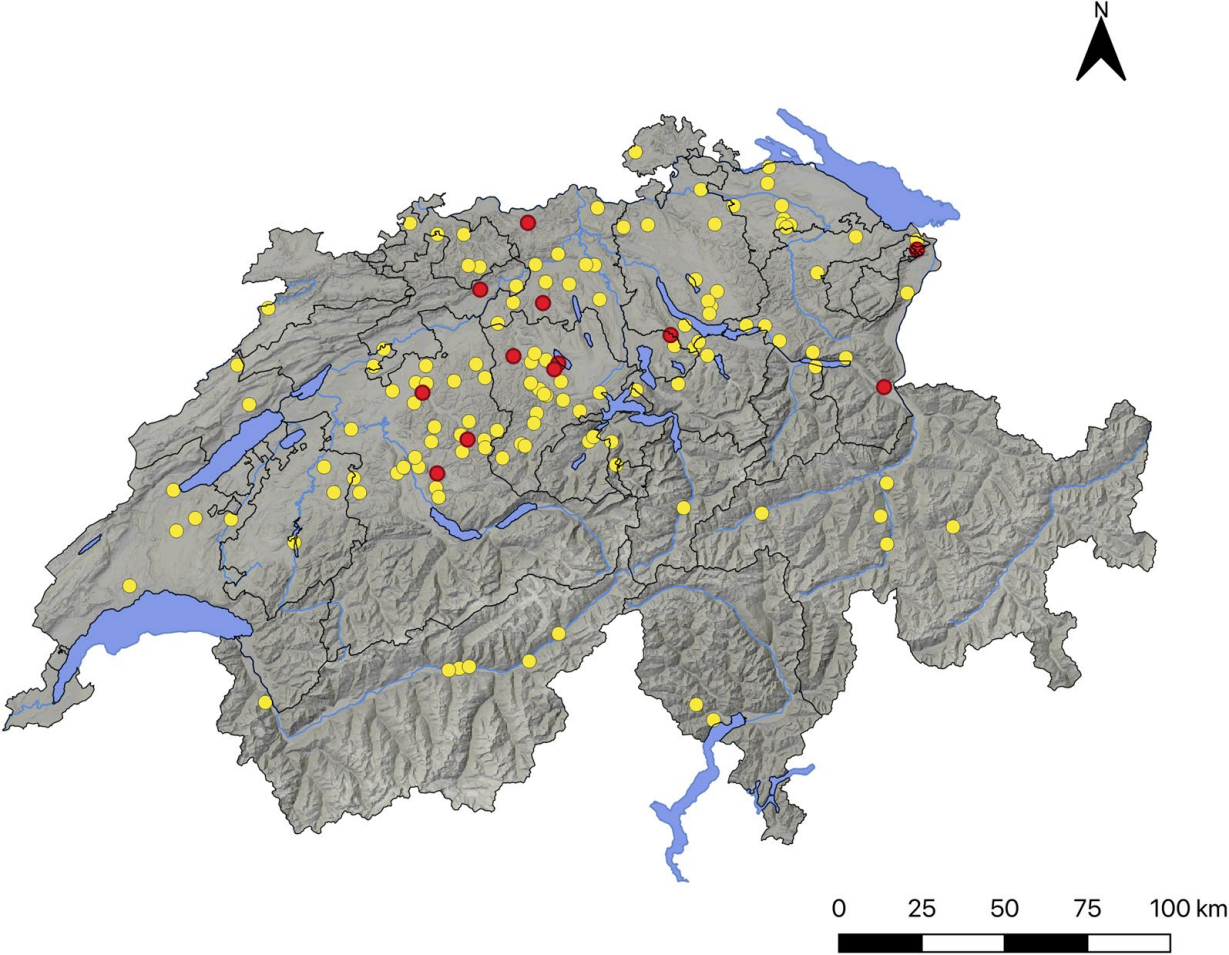
Map of Switzerland showing the distribution of South American camelid (SAC) breeding farms (*n* = 132) sampled in the study (all dots) and the farms in which *N. caninum* seropositive SAC were detected (red dots)

A total of 571 serum samples (i.e. 374 alpacas and 197 llamas) from 132 farms distributed along all 26 Swiss cantons was obtained (Fig. 1). The collected samples represented 8.6% of the total SAC population (*n* = 6619) and 10.2% and 6.7% of the total alpaca and llama populations (*n* = 3666 and 2953, respectively) in Switzerland at the beginning of sampling, respectively [29].

In order to assess putative risk factors for exposure, a standardized web-based questionnaire was prepared in three Swiss national languages (i.e. German, French and Italian) and subsequently sent to the participating farms *via* e-mail. The questionnaire contained general questions on the farm, animals (i.e. SAC species, breed, number of SAC in the farm, origin), management (i.e. type of water supply, type of feeding, feed storage, presence and number of cats and dogs (and especially of kittens and puppies < 6 months-old) in the farm during the last two years, access of cats/dogs to the stables and pasture, problems with rodents, presence of other animal species) and occurrence of abortions. Answering of the questionnaire was voluntary for all participants. All collected data, as well as the subsequent evaluations were processed confidentially.

### Control sera

In all serological tests, known *N. caninum* or *T. gondii* positive and negative control sera were included in addition to those provided with the kits.

For the evaluation of commercial *N. caninum* ELISAs and in-house *N. caninum* immunoblot, serial sera from two 1-year-old llamas, which had been intravenously (iv) inoculated either with 4.8 × 10E6 cell culture-derived *N. caninum* tachyzoites (Llama 1) or with 5 × 10E4 Vero cells (Llama 2) on day 0 of the experiment were used as positive and negative controls, respectively [30]. Serum samples from both animals were collected at 12 time points between days 0 and 52 after inoculation. While Llama 1 seroconverted against *N. caninum*, Llama 2 remained seronegative throughout the observation period [30]. Serum samples of both llamas obtained on day 52 post-inoculation were used as internal positive and negative controls in all test runs, in addition to the controls provided by the manufacturer.

For the evaluation of a commercial *T. gondii* ELISA and in-house *T. gondii* immunoblot, control sera from a naturally infected llama (Llama 2) (this llama, used as negative control in the experimental inoculation with *N. caninum* was seropositive for *T. gondii* at all samplings), five naturally infected alpacas (Alpaca 1-5) and three seronegative alpacas (Alpaca 6-8), previously tested at the Friedrich-Loeffler-Institut (FLI), Germany, by immunoblot were used. In all test runs positive (Llama 2) and negative (Alpaca 8) internal control sera were included in addition to the controls provided by the manufacturer. Additionally, in *N. caninum* tests, serum from a calf, experimentally inoculated with *N. caninum* tachyzoites was used [31].

### Serological tests

Since no validated serological tests for antibody detection against *T. gondii* or *N. caninum* in SAC were available, in the first phase of the study commercial ELISA kits were evaluated for their use in these animal species comparing their results with those of immunoblot tests.

### ELISA

In a first step, two commercially available ELISA kits: ID Screen® Toxoplasmosis Indirect (TOXO-MS; for dogs, cats, goats, sheep, cattle and pigs; ID.vet, Grabels, France) and ID Screen® Neospora caninum Indirect Multi-species (NCS-MS; for dogs, goats, sheep and cattle; ID.vet) were used for screening according to the manufacturerʼs instructions. In both kits, multi-species conjugates were used as secondary antibodies.

For each serum, a sample-to-positive ratio (S/P ratio) was calculated based on the optical density (OD) of the sample and the positive and negative controls of the kit according to the formula S/P% = (OD sample − OD negative control/ OD positive control − OD negative control) × 100. According to the manufacturer, animals with S/P% ≤ 40% are considered negative, inconclusive if 40% < S/P% > 50% and positive if S/P% ≥ 50%. Since these kits are not validated for SAC, the ELISA cut-offs were later re-adjusted based on immunoblot results.

In order to assess if the results obtained with NCS-MS ELISA were comparable with those from other kits, in a second step, all sera tested for antibodies against *N. caninum* by both NCS-MS ELISA and *N. caninum* immunoblot were re-tested by two additional ELISA kits: (i) ID Screen® Neospora caninum Indirect (NCS; indirect ELISA for detection of antibodies against *N. caninum* in serum, plasma or milk from cattle, sheep or goats; ID.vet), which uses an anti-ruminant conjugate; and (ii) ID Screen® Neospora caninum Competition (NCC; competitive ELISA for detection of antibodies against *N. caninum* in serum or plasma from cattle, sheep, goats, dogs or other susceptible species; ID.vet). The cut-offs indicated by the manufacturer for NCS ELISA were the same ones as for NCS-MS ELISA. In the Competition ELISA, for each sample the competition percentage (S/N%) was calculated according to the formula: S/N% = (OD sample/OD negative control) × 100. The recommended cut-offs by the manufacturer were: S/N% ≤ 50%: positive, doubtful if 50% < S/N% ≤ 60% and negative if S/N% > 60%. The Competition ELISA was performed with the overnight incubation protocol, as described by the manufacturer.

### Immunoblot

At the Institute of Parasitology, Vetsuisse Faculty Bern (IPB), in-house *T. gondii* tachyzoite surface antigen TgSAG1 (P30)-based and *N. caninum* tachyzoite-based immunoblots were performed to confirm ELISA results or to optimize the cut-offs of the commercial kits.

Pellets containing either 3 µg of native affinity-purified *T. gondii* TgSAG1 antigen (TOXO P30 B-10 Antigen, SR2B: Société de Recherche et de Réalisations Biotechnologiques, Avrillé, France) or 2.6 × 10E7 *N. caninum* tachyzoites were resuspended in 400 μl sample buffer [32], incubated for 5 min at 95 °C and 1000 *rpm* in a Thermomixer compact (Eppendorf), and electrophoresed in two precast polyacrylamide gels (Criterion^TM^ TGX Stain-Free^TM^ 4–20% precast gels for PAGE, Bio-Rad, Hercules, CA, USA) under non-reducing conditions along with a pre-stained protein standard (Precision Plus Protein^TM^ Dual Extra Standards, Bio-Rad). Subsequently, the antigens were electrophoretically transferred to nitrocellulose membranes (Trans-Blot Turbo^TM^, Bio-Rad), cut into stripes and blocked for 30 min with blocking solution (PBS with 0.05% (v/v) Tween 20 (Sigma, Munich, Germany) and 2% (v/v) fish gelatine (Serva Electrophoresis GmbH, Heidelberg, Germany)). Selected serum samples (according to ELISA results) were tested at 1:100 dilution in blocking solution and incubated at room temperature for 60 min. Afterwards, the stripes were washed 5 times during 5 min with 0.05% Tween 20-PBS solution. For detection of specific antibodies against *T. gondii*-SAG1 antigen or against *N. caninum*, the stripes were incubated with a rabbit anti-llama IgG (H + L) secondary antibody, HRP (Invitrogen, Thermo Fisher Scientific, Waltham, MA, USA) at 1:600 dilution in 0.05% Tween 20-PBS at room temperature for 60 min. Washing was performed as indicated above and followed by a 15–20 min incubation step with substrate solution (40 µl H_2_O_2_ (30% (v/v) and 30 mg 4-chloro-1-naphthol (Sigma-Aldrich) in 40 ml PBS, 20% (v/v) methanol). The reaction was stopped by addition of distilled water. For *T. gondii*, a serum reaction against the immunodominant antigen TgSAG1 visible as a sole band of a relative molecular mass of 30 kDa was recorded as positive. For *N. caninum*, a serum was considered positive when reaction against at least two out of five *N. caninum* relevant immunodominant antigens (i.e. 17, 29, 30, 33 and 37 kDa) were observed [30, 32]. If only one band was visible, the sample was considered inconclusive.

### ROC-analysis and optimization of ELISA cut-offs for SAC

The R software, version 3.5.3 [33] and the R package *optimal.cutpoints* were used to define optimal cut-offs for the commercial ELISA kits to detect antibodies against *T. gondii* (TOXO-MS) and *N. caninum* (NCS-MS and NCC) in SAC. For this, receiver operating characteristics (ROC) curve analysis was used, and relative to the immunoblot results the area under the ROC curve (AUC), diagnostic sensitivity, specificity, and positive and negative predictive values including 95% confidence intervals (95% CI) were determined. According to an arbitrary guideline, the area under the curve (AUC) was considered: non-informative (AUC = 0.5); low accurate (0.5 < AUC ≤ 0.7); moderately accurate (0.7 < AUC ≤ 0.9); highly accurate (0.9 < AUC < 1); or perfect (AUC = 1) [34].

To evaluate the agreement among different tests considering the ELISA cut-offs suggested by the manufacturer and the optimized cut-offs, an inter-rater agreement (kappa) was calculated [35] and kappa values (κ) were considered as follows: slight agreement (ĸ = 0–0.20); fair agreement (ĸ = 0.21–0.40); moderate agreement (ĸ = 0.41–0.60); substantial agreement (ĸ = 0.61–0.80); or almost perfect agreement (ĸ = 0.81–1.00) [36].

### Risk factor analysis

A risk factor analysis for *T. gondii* and *N. caninum* seropositivity was performed based on the information collected with the web-based questionnaire.

First, univariate models were performed considering the farm status (“seropositive” or “seronegative”) as dependent variables and the potential risk factors included in the questionnaire as independent variables. One farm was considered “seropositive” for *T. gondii* or *N. caninum* when at least one of the tested animals in the farm gave a positive serological result for these parasites. All risk factors were tested as categorical variables with the Pearsonʼs Chi-square test and the Likelihood Ratio. The null hypothesis (H0) aimed to prove the independence between the infection status and the corresponding risk factors (categorical variables).

Secondly, bivariable-multilevel-modelling (generalized linear mixed modelling fit by maximum likelihood (Laplace approximation)) was performed using R (http://www.R-project.org) version 3.3.5, by applying the package *lme4*. Because individual animals clustered in farms, “farm” was included as a random effect variable. Because in *T. gondii* seropositivity increased with age, “age” (in months) of individual animals had to be regarded as an important risk or effect-modifying explanatory variable. Thus, data on age of the animals were included into each of the bivariable models calculated. Animals, for which no birth date was available were excluded from the analysis. The Akaike information criterion (AIC) was used to characterize the relative model quality. The serological status (“seropositive” or “seronegative”) for *T. gondii* and *N. caninum* infections that were considered in the analyses were those obtained by TOXO-MS and NCS-MS ELISA, respectively, when the optimized cut-offs were used.

## Results

### ID Screen® Toxoplasmosis indirect ELISA (TOXO-MS)

Considering the thresholds suggested by the kit’s manufacturer, 468/571, 4/571 and 99/571 of the tested animals showed a positive, inconclusive and negative result for *T. gondii*, respectively.

Llama 1, experimentally inoculated with *N. caninum* remained seronegative for *T. gondii* throughout the experimental period (ELISA S/P values between 9.34–17.76%). Llama 2, experimentally inoculated with Vero cells and used as a negative control for *N. caninum*, always showed ELISA S/P values above 100% (106.41–129.12%) for *T. gondii* and was therefore considered as naturally infected with this parasite. All five known naturally infected alpacas (Alpaca 1-5) used as positive controls also gave positive results in the *T. gondii* ELISA (S/P 122.81–207.11%). The two negative control alpacas (Alpaca 6-8) tested also negative in the commercial ELISA with values well below the cut-off suggested by the manufacturer (S/P 4.1%, 3.05% and 10.08%, respectively). When the cut-off was re-adjusted based on ROC analysis considering the immunoblot results (Table 3; i.e. cut-off = S/P 36.2%, relative sensitivity = 94.5%, relative specificity = 97.9%), the general seroprevalence at the animal level was 83.2% (475/571, i.e. 82.3% (308/374) of the alpacas and 84.8% (167/197) of the llamas were seropositive for *T. gondii*) and the seroprevalence at the farm level was 99.2% (131/132). Only in one of the 132 participating farms all tested animals (4/4) were seronegative for *T. gondii*.

**Table 3:** Results of receiver operating chraracteristics (ROC) analysis

ELISA Kit	Optimized ELISA cut-off	AUC (95% CI)	% Relative sensitivity (95% CI)	% Relative specificity (95% CI)	PPV % (95% CI)	NPV % (95% CI)
TOXO-MS	≥ 36.2 S/P%	0.97 (0.94–1.00)	94.5 (87.6–98.2)	97.9 (92.6–99.7)	97.7 (92.0–99.3)	94.9 (88.5–99.4)
NCS-MS	≥ 34.9 S/P%	0.96 (0.91–1.01)	89.3 (71.8–97.7)	100.0 (95.4–NaN)	100 (86.9–100)	96.3 (88.8–NaN)
NCC	≤ 78.6 S/N%	0.97 (0.92–1.01)	92.9 (76.5–99.1)	97.4 (91.0–99.7)	92.9 (77.7–99.1)	97.4 (90.5–99.7)

*Notes*: Relative accuracies of commercial ELISA kits used for detection of antibodies against *Toxoplasma gondii* (i.e. ID Screen® Toxoplasmosis Indirect ELISA) and *N. caninum* (i.e. ID Screen® Neospora caninum Indirect Multi-species ELISA and ID Screen® Neospora caninum Competition ELISA) in South American camelids, in relation to *N. caninum* and *T. gondii* immunoblots

*Abbreviations*: TOXO-MS, ID Screen® Toxoplasmosis Indirect; NCS-MS, ID Screen® Neospora caninum Indirect Multi-species ELISA; NCC, ID Screen® Neospora caninum Competition; S/P%, sample-to-positive ratio; S/N%, competition percentage; AUC, area under the ROC curve; CI, confidence interval; TP, true positives; FP, false positives; TN, true negatives; FN, false negatives; PPV, positive predictive value; NPV, negative predictive value; NaN, upper limit of the confidence interval could not be computed

### *Toxoplasma gondii* immunoblot

**Fig. 2:**
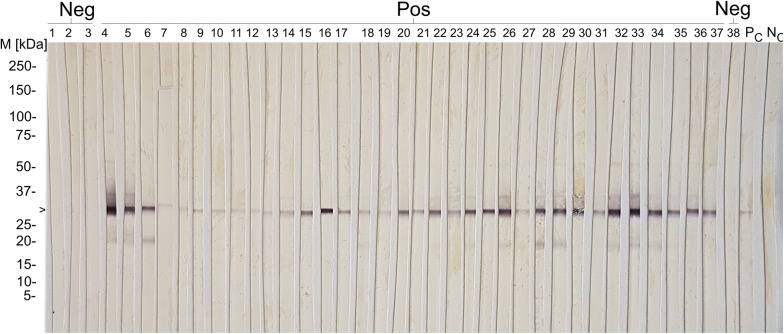
Immunoblot results for antibodies against *T. gondii* in Swiss South American camelids (SAC). *Abbreviations*: M, molecular marker; Neg, seronegative Swiss SAC field samples; Pos, seropositive Swiss SAC field samples; PC, positive control (Llama); NC, negative control (Llama); >, 30 kDa band corresponding to SAG-1 *T. gondii* tachyzoite surface antigen. Samples recognizing the 30 kDa antigenic band were considered positive

All control animals (*n* = 9) and selected field serum samples (*n* = 177) based on the ELISA results using the manufacturer’s cut-offs were tested by immunoblot, i.e. all negative samples (*n* = 97; S/P 1.70–39.13%), all samples with inconclusive results (*n* = 4; S/P 42.30–49.93%) and all positive samples with S/P values of 50.83–101.07% (*n* = 26), as well as five groups of five samples each, randomly selected within arbitrarily defined intervals (i.e. group 1: S/P 120.14–125.07%; group 2: S/P 149.93–152.30%; group 3: S/P 170.74–172.33 %; group 4: S/P 190.29–191.09%; group 5: S/P 220.00–221.70%); and the 25 serum samples with the highest S/P values ​​(S/P 247.04–284.23%) (Fig. 2).

The *T. gondii* ELISA kit showed almost perfect agreement with immunoblot results (considering the manufacturer’s cut-off, weighted k = 0.887; considering the optimized cut-off, k = 0.925) (Table 4). All but two (86/88) of the selected animals with positive ELISA results and all four samples with inconclusive results yielded positive results in immunoblot. Eight out of 102 serum samples that were first considered negative in ELISA (S/P < 40%) showed a positive result in the immunoblot. The *T. gondii* naturally infected llama (Llama 2) and all five naturally infected alpacas (Alpaca 1-5) showed immunoblot positive reactions, and the negative control alpacas (Alpaca 6-8) were confirmed as seronegative.

**Table 4:** Inter-rater agreement (Kappa value) between ELISA kits for detection of antibodies against *T. gondii* (ID Screen® Toxoplasmosis Indirect) or *N. caninum* (ID Screen® Neospora caninum indirect multi-species and ID Screen® Neospora caninum Competition) and immunoblot, and between ELISA kits (ID Screen® Neospora caninum Indirect Multi-species and ID Screen® Neospora caninum Competition)

		TOXO-MS (manufacturer’s cut-offs)				TOXO-MS (optimized cut-off)				
		Positive	Doubtful	Negative	Total	Positive	Doubtful	Negative	Total	
*T. gondii* immunoblot	Positive	80	3	8	91	86	0	5	91	
	Doubtful	0	0	0	0	0	0	0	0	
	Negative	1	0	94	95	2	0	93	95	
	Total	81	3	102	186	88	0	98	186	
	Kappa (κ)	0.873				0.925				
	SE of κ	0.035				0.028				
	95% CI	0.804–0.941				0.870–0.979				
	Weighted κ	0.887				0.925				

		NCS-MS (manufacturer’s cut-offs)				NCS-MS (optimized cut-off)				
		Positive	Doubtful	Negative	Total	Positive	Doubtful	Negative	Total	
*N. caninum* immunoblot	Positive	19	5	4	28	25	0	3	28	
	Doubtful	0	0	2	2	0	0	2	2	
	Negative	0	0	76	76	0	0	76	76	
	Total	19	5	82	106	25	0	81	106	
	Kappa (κ)	0.739				0.879				
	SE of κ	0.066				0.052				
	95% CI	0.610–0.868				0.777–0.981				
	Weighted κ	0.806				0.901				

		NCC (manufacturer’s cut-offs)				NCC (optimized cut-off)				
		Positive	Doubtful	Negative	Total	Positive	Doubtful	Negative	Total	
*N. caninum* immunoblot	Positive	21	5	2	28	26	0	2	28	
	Doubtful	1	1	0	2	2	0	0	2	
	Negative	0	0	76	76	1	0	75	76	
	Total	22	6	78	106	29	0	77	106	
	Kappa (κ)	0.819				0.884				
	SE of κ	0.055				0.049				
	95% CI	0.710–0.927				0.788–0.980				
	Weighted κ	0.876				0.905				

	A	NCC (manufacturer’s cut-offs)				B	NCC (optimized cut-off)			
		Positive	Doubtful	Negative	Total		Positive	Doubtful	Negative	Total
NCS-MS (A: manufacturer’s cut-off B: optimized cut-off)	Positive	18	1	0	19		26	0	0	26
	Doubtful	3	2	0	5		0	0	0	0
	Negative	1	3	78	82		3	0	77	80
	Total	22	6	78	106		29	0	77	106
	Kappa (κ)	0.807					0.926			
	SE of κ	0.060					0.042			
	95% CI	0.689–0.925					0.845–1.000			
	Weighted κ	0.876					0.926			

TOXO-MS: ID Screen® Toxoplasmosis Indirect; NCS-MS: ID Screen® Neospora caninum Indirect Multi-species ELISA; NCC: ID Screen® Neospora caninum Competition; SE: Standard Error; CI : Confidence Interval

### ID Screen® *Neospora caninum* indirect multi-species ELISA (NCS-MS)

**Fig. 3:**
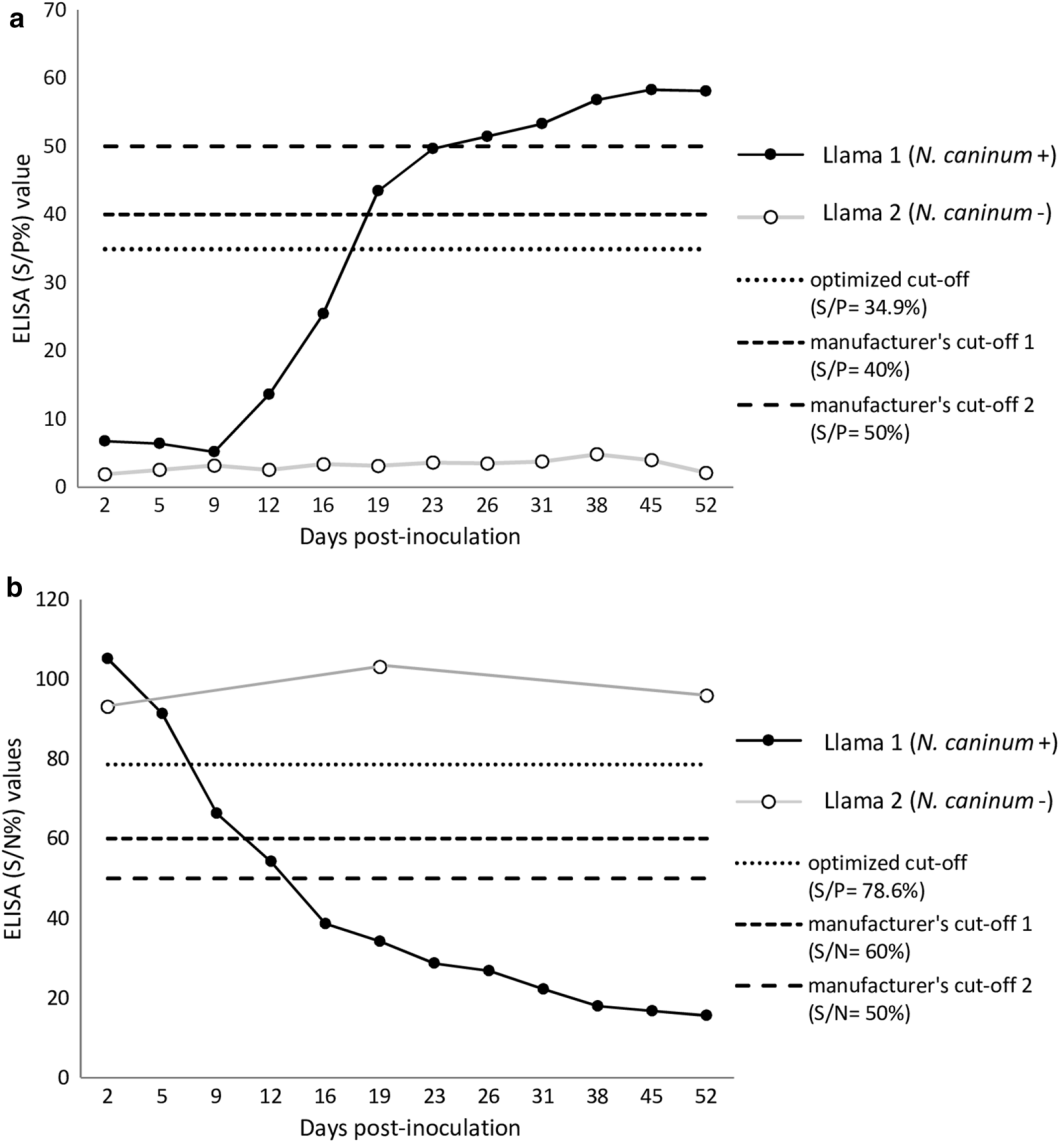
ELISA results for antibodies against *N. caninum* on serum samples from a llama experimentally inoculated with 4.8 × 10E6 cell culture-derived *N. caninum* tachyzoites (Llama 1) and from a control llama inoculated with 5 × 10E4 Vero cells (Llama 2) [30]. **a** ID Screen® *Neospora caninum* indirect multi-species, S/P% = sample-to-positive ratio, calculated based on the OD (optical density) of the sample and the positive and negative controls of the kit according to the formula S/P% = (OD_sample_ − OD_negative control_/OD_positive control_ − OD_negative control_) × 100. **b** ID Screen® *Neospora caninum* Competition, S/N% = competition percentage, calculated according to the formula: S/N% = (OD_sample_/OD_negative control_) × 100

Considering the cut-offs proposed by the manufacturer for other animal species, 14/571, 3/571 and 554/571 out of the tested SAC field samples yielded a positive, inconclusive and negative result for *N. caninum*, respectively. Considering these cut-offs, the llama experimentally infected with *N. caninum* (Llama 1) showed inconclusive ELISA values between 19–23 dpi (S/P 43.45–49.66%) and positive values (S/P ≥ 51.45%) from 26 dpi until the end of the observation period (52 dpi). The negative control animal (Llama 2) showed S/P values below the cut-off (1.89–4.82%) at all samplings (Fig. 3).

In order to adapt the kit for testing SAC, the ELISA cut-off was recalculated by a ROC-analysis based on the immunoblot results (Table 3). With an optimized cut-off (i.e. cut-off = S/P 34.9%, relative sensitivity = 89.3%, relative specificity = 100%), 3.15% (18/571) of the sampled SAC tested positive for *N. caninum.* According to the SAC species, the estimated seroprevalences in alpacas and llamas were 3.5% (13/374) and 2.5% (5/197), respectively, and the seroprevalence at the farm level was 9.1% (12/132). Considering the optimized ELISA cut-off, seroconversion of the experimentally infected llama could be already observed at 19 dpi. (Fig. 3).

The 18 seropositive animals for *N. caninum* in NCS-MS ELISA were 13 alpacas and five llamas, 17 animals older than 36 months and one animal of unknown age, 13 females and five males, and derived from 12 different farms. In nine of the farms only one animal/ farm (i.e. five llamas and four alpacas) tested positive, and in three farms 4/5, 3/4 and 2/5 of the tested alpacas were seropositive. The mean ELISA S/P values of the 18 seropositive animals was 76.4%. All *N. caninum* seropositive animals were also *T. gondii-*seropositive.

### *Neospora caninum* immunoblot

**Fig. 4:**
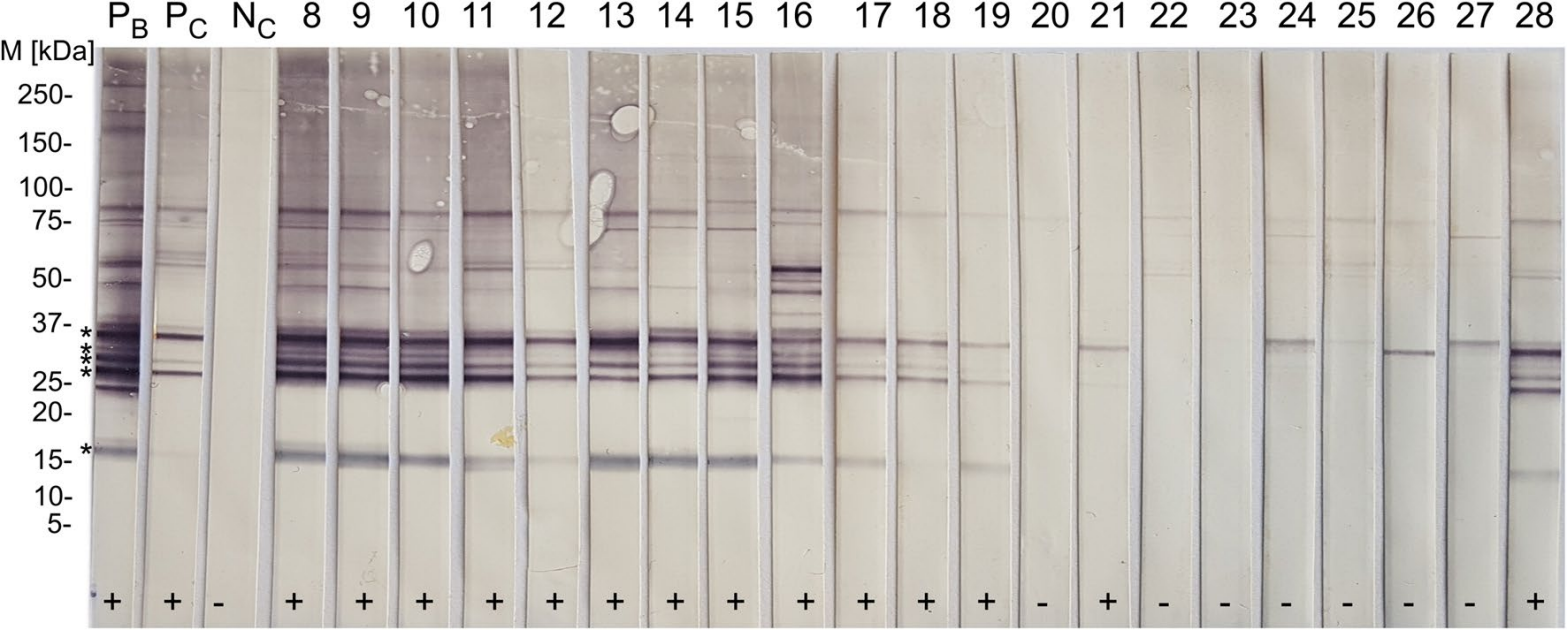
Immunoblot results for antibodies against *N. caninum* on field serum samples from Swiss South American camelids (SAC). *Abbreviations*: M, marker; PB, positive control (bovine); PC, positive control (Llama 1); NC, negative control (Llama 2); samples 8–28, Swiss SAC field serum samples; +, positive immunoblot result; −, negative immunoblot result. Bands corresponding to immunodominant *N. caninum* antigens (17, 29, 30, 33 and 37 kDa) are indicated with *. Samples recognizing two or more immunodominant antigens were considered positive

Selected field samples (*n* = 92) and the control animals (Llama 1 and 2) were tested by *N. caninum* immunoblot. Based on the results in the NCS-MS ELISA, all samples with an S/P ≥ 9% (*n* = 59) and further 33 randomly selected samples within the S/P range 1–8.31% were further tested by immunoblot. All 14 animals, which were classified as positive using the cut-off recommended by the manufacturer, as well as the three animals classified as inconclusive, tested positive in immunoblot (Fig. 4). A further animal with S/P value 34.9% yielded a clear positive result. An additional sample with S/P value of 14.15% in the ELISA also reacted against the immunodominant *N. caninum* antigens. This was a llama from a farm in which another *N. caninum* seropositive animal (by both ELISA and immunoblot) had been detected. Besides, also two alpacas from a same farm with ELISA S/P values of 5.39 and 9.99 were positive in the immunoblot.

**Fig. 5:**
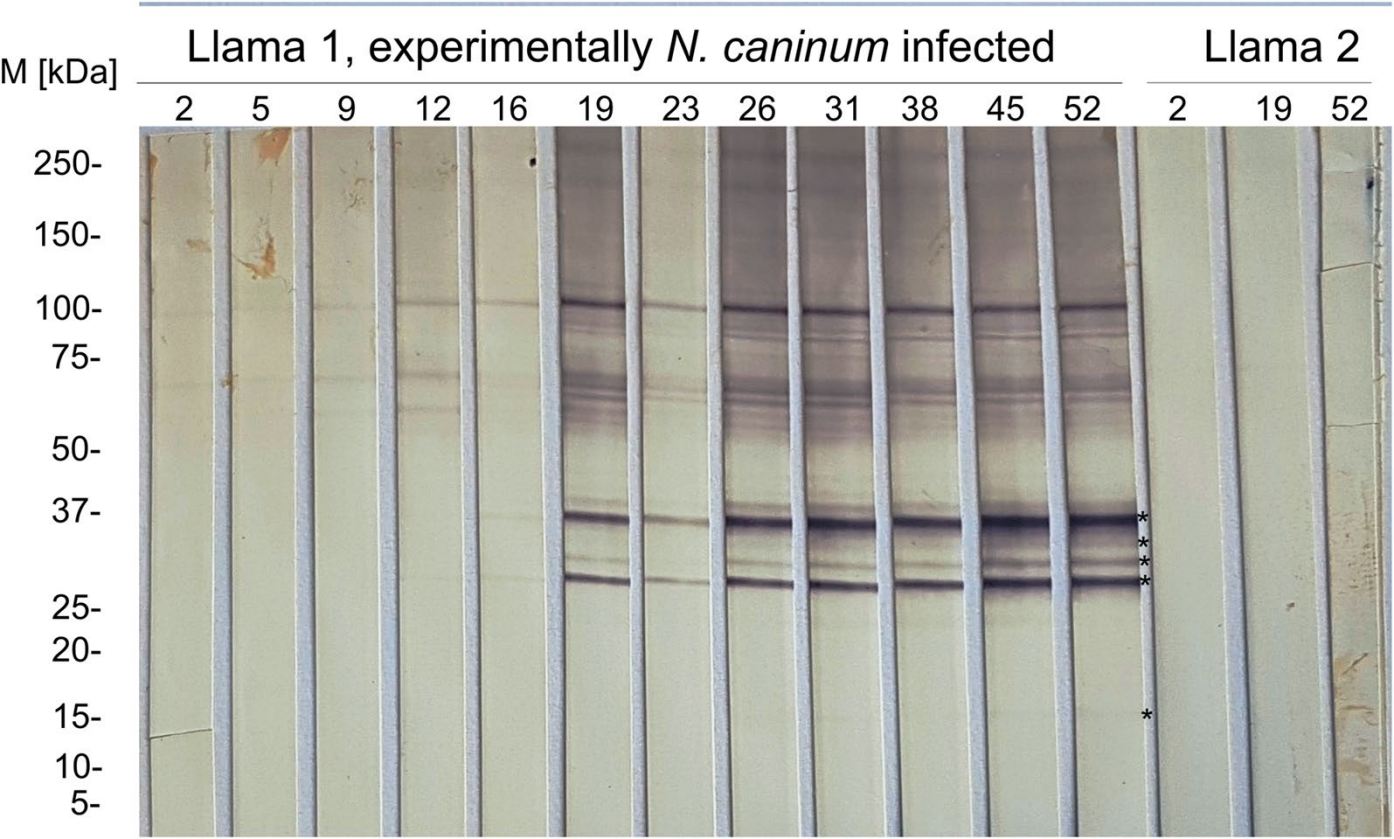
Immunoblot results for antibodies against *N. caninum* on serum samples from a llama experimentally inoculated with 4.8 × 10E6 cell culture-derived *N. caninum* tachyzoites (Llama 1) and from a seronegative control llama inoculated with 5 × 10E4 Vero cells (Llama 2). *Abbreviations*: M, marker; kDa, kilodaltons; 2–52 (indicated above), days post-inoculation (dpi). Bands corresponding to immunodominant *N. caninum* antigens (17, 29, 30, 33 and 37 kDa) are indicated with an asterisk. In Llama 1, reaction against the 29 kDa antigen is observed at 12 dpi. From 16 to 52 dpi also further immunodominant antigens are recognized. Samples recognizing two or more immunodominant antigens were considered positive. Llama 2 did not recognize any of the *N. caninum* immunodominant antigens during the observation period

The llama experimentally inoculated with *N. caninum* tachyzoites (Llama 1), showed reaction against the 29 kDa immunodominant antigen already at 12 dpi (Fig. 5), and against two or more immunodominant *N. caninum* antigens (17, 29, 30, 33 and 37 kDa) from 16 dpi. The *N. caninum* seronegative control animal (Llama 2) did not show any reaction against *N. caninum* antigens throughout the whole observation period (tested at 2, 19 and 52 dpi) (Fig. 5).

### ID Screen® *Neospora caninum* Competition ELISA (NCC)

All sera tested by both NCS-MS ELISA and immunoblot were subsequently tested by NCC ELISA and the cut-off was optimized for SAC based on immunoblot results, as indicated above. By ROC analysis, a cut-off of S/N% ≤ 78.6 was suggested (Table 3). With the optimized cut-off, NCC ELISA evidenced a relative sensitivity of 92.9% and a relative specificity of 97.4% respect to the *N. caninum* immunoblot, and an almost perfect agreement with immunoblot and NCS-MS results (Table 4). With the competitive ELISA (considering the optimized cut-off), seroconversion in Llama 1 (experimentally inoculated with *N. caninum* tachyzoites) could already be detected at 9 dpi, with positive ELISA values until the end of the study at 52 dpi. If the cut-offs suggested by the manufacturer were considered, seroconversion would have occurred 16 dpi, with doubtful values at 12 dpi (Fig 3).

### ID Screen® *Neospora caninum* indirect ELISA (NCS)

All analysed sera, including those from animals positive in *N. caninum* immunoblot and serial samples from the llama experimentally infected with *N. caninum* gave negative results (ELISA values similar to those from the negative controls) in NCS ELISA using an anti-ruminant conjugate.

### ROC-analysis and optimization of ELISA cut-offs for SAC

The relative accuracies of NCS-MS, NCC and TOXO-MS ELISAs in relation to *N. caninum* and *T. gondii* immunoblots, respectively, are provided in Table 3. The statistical analyses suggested a cut-off of S/P 34.9% for NCS-MS, 78.6% S/N for NCC, and 36.2% S/P for TOXO-MS ELISA (Table 3). The inter-rater agreement (kappa value) between all three ELISAs and the respective immunoblots and between NCS-MS and NCC ELISA are provided in Table 4. The agreement of all three tested ELISAs with immunoblot was almost perfect, being higher when the adapted cut-offs were considered. Further, also the agreement between both *N. caninum* ELISAs (NCS-MS and NCC) was almost perfect (Table 4).

### Questionnaire and risk factor analysis

A total of 41 out of 132 (31.1%) farms responded to the web-based questionnaire. These farms accounted for 189 out of the 571 (33%) animals sampled in the survey. Twenty-two farms bred exclusively alpacas, 12 farms exclusively llamas and seven farms bred both species. Considering only the responding farms and the adapted ELISA cut-offs, the seroprevalences for *T. gondii* and *N. caninum* at the farm level were 100% (*n* = 41/41) and 14.6% (*n* = 6/41), respectively, and at the animal level 77.8% (*n* = 147/189) and 5.8% (*n* = 11/189), respectively. Regarding the SAC species, 75.8% (91/120) of the alpacas and 81.2% (56/69) of the llamas were seropositive for *T. gondii* and 6.7% (8/120) of the alpacas and 4.3% (3/69) of the llamas were seropositive for *N. caninum*.

All 41 responding farms bred Swiss SAC (born at the same farm or purchased from other Swiss farms). Only five farms (12.2%) had purchased few animals abroad (i.e. Peru, Chile, USA, Australia, New Zealand and Germany). None of the tested animals from those five farms evidenced antibodies against *N. caninum*.

Thirty-five (85.4%) of the farms reported presence of cats (one cat up to >10 cats) in the last two years, and kittens had been present in eight of these farms (22.9%). Either own or foreign/neighbouring cats had access to the pasture and stable in 34 (97.1%) and 33 (94.3%) of these farms, respectively. Own dogs (up to four dogs) were present in 22/41 (56.7%) of the farms during the last two years and two (9.1%) of those farms had puppies. Own dogs had access to the stable and pasture in 21 (95.4%) of those 22 farms and foreign/neighbouring dogs were seen in at least 11 farms. Seven (17%) of the farms had problems with rodents, and 34/41 (82.9%) of the farms reported breeding other animal species (i.e. cattle, sheep, goats, equids, pigs, poultry, rabbits, quails, and fallow deer) besides SAC. Eleven (26.8%) of the participating farms experienced abortions (up to five abortions) during the last two years and only one of the farms submitted the aborted foetuses for investigation, but no abortion cause could be detected.

Since all responding farms were *T. gondii*-seropositive (i.e. all 41 farms had at least one seropositive animal), no risk analysis for *T. gondii* at the farm level was possible. By generalized linear mixed modelling considering the individual serological status, the variable “age” (in months) was identified as putative risk factor for infection with *T. gondii* (Table 5) and female animals had a significantly increased risk of being *T. gondii-*seropositive (Table 5). The variable “absence of cats in the farm during the last two years” was identified as a protective factor (Table 5).

**Table 5:** Fixed effects in generalized linear mixed models to determine potential risk factors for *Toxoplasma gondii*-seropositivity in Swiss South American camelids

Model (AIC, model fit)	Variable	Odds ratio (95% CI)	*z-*value	*P*-value
1 (155.9)	Intercept	1.090 (0.390–3.070)	0.170	0.864644
	Age (months)	1.020 (1.010–1.040)	3.602	0.000316***
2 (153.4)	Intercept	0.607 (0.209–1.760)	− 0.919	0.358139
	Age	1.021 (1.009–1.030)	3.497	0.000471***
	Sex: male (ref.)			
	Sex: female	2.924 (1.111–7.690)	2.174	0.029711*
3 (152.4)	Intercept	1.228 (0.460–3.278)	0.410	0.681820
	Age	1.024 (1.011–1.036)	3.707	0.000210***
	Cat, past 2 years (ref.)			
	No cat, past 2 years	0.214 (0.055–0.828)	− 2.233	0.025530*
4 (149.3)	Intercept	0.692 (0.255–1.880)	− 0.722	0.470200
	Age	1.021 (1.009–1.033)	3.540	0.000400***
	Sex: male (ref.)			
	Sex: female	2.941 (1.164–7.426)	2.282	0.022500*
	Cat, past 2 years (ref.)			
	No cat, past 2 years	0.232 (0.066–0.809)	− 2.293	0.021900*

*Notes:* Data were analysed by bivariable generalized linear mixed modelling including “Age” (months) as effect modifier and “Farm” as random effects variable in modelling *T. gondii*-seropositivity. The Akaike information criterion (AIC) was used to characterize the relative model quality

*Abbreviation*: ref., reference

*P ≤ 0.05, **P ≤ 0.01, ***P < 0.001

None of the examined variables could be classified as a risk factor for *N. caninum* seropositivity, as the observed differences were not statistically significant. Moreover, modelling considering the infection status of the individual animals was not possible, because only 11 animals of six out of 41 farms that had answered the questionnaire tested positive, with four of these farms in which only a single animal tested positive. The relation of the putative risk factors with seropositivity to *N. caninum* at the farm level is displayed in Additional file 1: Table S1.

## Discussion

The multi-species *T. gondii* and *N. caninum* ELISA kits used in this study (i.e. TOXO-MS and NCS-MS) were not previously validated for their use on SAC; therefore, the results obtained on SAC field samples were further confirmed by immunoblot, and control sera with known positive or negative serological status for *T. gondii* and *N. caninum* infections were included in the analyses. The immunoblot method was chosen as “gold standard for comparison” to estimate the cut-off of the kits for testing SAC and their relative diagnostic characteristics considering the optimized cut-offs. Subsequently, two further ELISA kits for detection of antibodies against *N. caninum* (i.e. NCC and NCS) were additionally evaluated on samples with known immunoblot results. With the optimized cut-offs, TOXO-MS, NCS-MS and NCC ELISAs had almost perfect agreement with immunoblot results (Table 4). Moreover, an almost perfect agreement was also observed between NCS-MS and NCC ELISAs (Table 4). On the other hand, a new version of the indirect ELISA (NCS), which uses an anti-ruminant conjugate, proved not to be suitable for testing SAC because it did not detect animals which tested seropositive for *N. caninum* antibodies both by immunoblot and by the two further tested *N. caninum* ELISAs. This kit differs from NCS-MS only in the used conjugate.

Almost nothing is known about the epidemiological situation or the clinical significance of *T. gondii* and *N. caninum* infections in SAC in Europe. Differences in the type of husbandry, possibility of contact with definitive hosts, geographical factors and/or occurrence of co-infections with other agents might influence the occurrence and/or clinical significance of these protozoan infections in Europe. Regarding prevalence studies on *T. gondii* infection, only few countries (i.e. Chile, Argentina, USA, Germany and Czech Republic) besides Peru (where several regional surveys have been carried out (Table 1)), investigated the occurrence of *T. gondii* antibodies in SAC. In Peru seroprevalences between 3.0 and 44.5% in alpacas, 8.6 and 44.2% in llamas and 2.6 and 5.5% in vicunas were reported (Table 1). In Argentina, a seroprevalence of 14.3% was reported in llamas from the North Western region by IFAT [37], and in Chile, prevalences of 7.2–11.8% in alpacas [38, 39] and 43.3% in llamas [39] were observed. In the USA, seroprevalences of 37.5 and 33.5% by MAT were observed in 16 alpacas [40] and 283 llamas [41], respectively. In Europe, antibodies against *T. gondii* were previously detected in a few SAC in Germany [30] and Czech Republic [42] by immunoblot, ELISA and/or IFAT. In this study, a general seroprevalence of 83.2% (475/571) in SAC living in Switzerland was revealed, which corresponded to seroprevalences of 82.3% (308/374) and 84.8% (167/197) in alpacas and llamas, respectively.

Seropositive animals derived from 131 out of 132 (99.2%) sampled farms. Although due to differences in the serological techniques and cut-offs used in the different surveys, a direct comparison of some studies is not always possible, the here observed prevalences are much higher than those reported worldwide so far (Table 1). This is in agreement with the high (i.e. 80.7%) *T. gondii* seroprevalence observed in Swiss ewes by P30-ELISA on meat juice [43]. The higher seroprevalence in SAC in Switzerland may be related to a more intensive management than in other geographical regions, enabling a closer contact with human premises and with domestic cats, which contaminate the pastures with their faeces. South American camelids often share grazing lands with sheep, being exposed to the same infection sources. Reported frequencies of domestic cats shedding *T. gondii* oocysts in Switzerland were between 0.4 [44] and 0.6% [45], which is probably > 10 times higher to that of dogs shedding *N. caninum* oocysts. Moreover, the number of owned cats living in Switzerland is approximately three times higher than that of owned dogs (in year 2018: 1,634,000 cats *vs* 506,000 dogs) [46], what can account for a higher environmental contamination with *T. gondii* oocysts.

Knowledge on the risk factors for infection is essential to make decisions on possible intervention measures. In ruminants, the main routes of infection with *T. gondii* include the ingestion of oocysts contaminating feed, water or the environment, and secondary, transplacental transmission [47]. In our study, the variables “age” and “female sex” were identified by multivariable statistical analysis as putative risk factors for *T. gondii* infection, while “absence of cats in the farm during the last two years” was suggested as a protective factor. The variable age was identified as a risk factor for *T. gondii* infection in numerous studies on different livestock species including small ruminants, cattle, equids, pigs and chicken [47]. In studies on SAC from Peru [30, 48–50], Chile [39] and Germany [30], it was observed that the seroprevalence of *T. gondii* infection increased with age, suggesting that most infections would occur postnatally. Accordingly, also in this study the variable age was estimated as a putative risk factor for infection (here also referred to as effect modifying variable in statistical analysis), and this can be explained, as older animals had more often the possibility of being exposed to *T. gondii* oocysts after birth than younger ones. While the variable “female sex” was suggested as putative risk factor for *T. gondii* seropositivity in few studies on small ruminants, pigs and equids; in most of the reported studies on livestock there was no statistical differences in the seroprevalence between both sexes [47]. It has to be considered that this apparent association might mask underlying factors such as differences in the way both sexes are reared more than to specific differences in susceptibility [47]. While in two studies from Peru, no statistical differences were observed in the seroprevalence of *T. gondii* infection between female and male alpacas [49, 50], in a further study it was found that female sex was a risk factor for acquiring toxoplasmosis in alpacas, but not in llamas [48]. The authors hypothesized that this could be due to differences in management, as llamas and male alpacas were pastured in cold, dry regions above 4000 m above sea level, and female alpacas were kept in lower, wet areas, what could account for a longer survival of *T. gondii* oocysts. Also in our study, female sex seemed to be a risk factor for *T. gondii* infection. In Switzerland, males are often held in “male herds”, separated from the females; however, there are no big differences in the general management of both sexes that could account for a higher exposure to the parasite in females. While different experimental studies suggested a higher susceptibility of female mice and guinea pigs to infection with *T. gondii* [47], it is not known if this also applies to SAC.

Further, the variable “absence of cats in the farm during the last two years” was identified as a “protective factor” for *T. gondii* infection. Cats, as definitive hosts of *T. gondii,* are responsible for environmental contamination with oocysts shed with their faeces, which may survive for several months (usually < 2 years) in the environment, and sporulated oocysts represent the main source of infection for ruminants [47]. Therefore, reporting an absence of cats on farms would account for lower chances of contamination of farmland, feed or water with oocysts.

The potential role of *T. gondii*-infected SAC meat as a source of infection for humans has been barely investigated and more studies are needed to better understand the correlation between seropositivity and presence of viable parasites in the tissues as well as to define the predilection sites of *T. gondii* in SAC. Recently, *T. gondii* was isolated from heart or muscle tissues from two out of six seropositive alpacas by bioassay in mice in the USA [40]. Meat from SAC represents an important food source for the Andean population in South America, where it is consumed as different local dishes not only cooked (e.g. as chupe, locro, pachamanca) but also raw in different preparations (e.g. as charqui, sausages or ceviche) [51]. In recent years, due to the higher protein and lower fat and cholesterol contents compared with meat from other livestock species, SAC meat has become an interesting kind of alternative red meat both for the local and international kitchen [52]. In Switzerland there are at least 15 listed providers of SAC meat and meat products [23]. SAC meat represents a gourmet dish, and it is often cooked only shortly at low temperature and consumed undercooked or raw as dried meat or sausages [23]. Given the high *T. gondii* seroprevalence observed, SAC meat might represent a potential infection source for humans if no treatment for parasite inactivation (e.g. cooking with core temperature > 67 °C, freezing at < − 12 °C for >2 days) [1] is carried out.

Surveys based on the presence of *N. caninum* antibodies in blood sera from SAC have been scarcely performed worldwide and data from only few countries (i.e. Peru, Argentina, Australia, Germany and Czech Republic) are available (Table 2). Most of the studies were performed in Peru, mainly in the Andes region, and revealed seroprevalences of 0.3–42.4% in alpacas, 0–23.3% in llamas and 0–1% in vicunas (*Vicugna vicugna*) by various serological tests (Table 2). In Argentina, a study on 308 llamas from the North-Western region revealed a seroprevalence of 1% by IFAT [7]. Outside South America, only studies involving few animals were performed and the observed seroprevalences were low (≤ 3%) [24, 30, 53]. In Europe, only two small serological studies have been published to date [30, 42]. *Neospora caninum* infection could not be serologically detected in any of 32 SAC by immunoblot in Germany [30], and it was diagnosed in one out of 9 SAC in the Czech Republic by ELISA and IFAT [42]. Accordingly, also in the present study low seroprevalences of *N. caninum* infection were observed. While most serosurveys for *N. caninum* and *T. gondii* infections were performed at local or regional levels, this study aimed to reveal the epidemiological situation of these protozoan infections at a nationwide level. Antibodies against *N. caninum* were detected by ELISA and confirmed by immunoblot in 3.15% (18/571) of the tested animals and in 9.1% (12/132) of the farms (Fig. 1). Considering the SAC species, 3.5% (13/374) of the alpacas and 2.5% (5/197) of the llamas tested in Switzerland were seropositive. Seropositive animals derived from 12 farms localized in seven Swiss cantons, showing a nationwide distribution of the parasite (Fig. 1). At least 12 out of the 18 seropositive animals derived from farms (*n* = 6) that reported not having imported animals. This suggests that the animals may get infected (horizontally or vertically) in Switzerland. For the remaining six animals, no information about a possible imported origin was known. In cattle, vertical transmission was considered the most important route of *N. caninum* infection, and it was shown that the parasite can persist in the herd during several generations even without further participation of the definitive host [54]. However, nothing is known about the efficiency of vertical transmission of *N. caninum* in SAC or about the meaning of horizontal transmission through the ingestion of sporulated oocysts shed by canids in these animal species. In Peru, no significant association was found between seropositivity to *N. caninum* in SAC and age [55, 56] or geographical region [57], suggesting that the infection might be mainly transmitted vertically [57]. Apart from some regional studies from Peru, in which higher seroprevalences of infection were reported (17.9–42.4 % in alpacas and 18.4–23.3% in llamas) [58, 59], only low seroprevalences were observed in studies outside South America, suggesting that only few animals had contact with the parasite. In Europe, *N. caninum* oocysts were detected in the faeces of only few dogs [60–64], with prevalences < 0.05% [63], suggesting that the environmental contamination with oocysts might be low. Nevertheless, acute infections with *N. caninum* oocysts were considered responsible for epidemic abortion outbreaks in several cattle farms in the region, showing that under certain epidemiological situations focal contamination with *N. caninum* oocysts may occur [65, 66].

It is believed that *T. gondii* and *N. caninum* infections in SAC are generally subclinical, and accordingly, serologically positive animals in this study did not show any clinical signs. However, in recent years a case of generalized fatal toxoplasmosis was described in a 13-month-old llama in the USA. Due to the character of the lesions, which included gastritis and enteritis, it was assumed that the animal became infected through ingestion of oocysts contaminating feed or water [21]. Therefore, also potential clinical aspects of this infection should be considered. Besides, *T. gondii* and *N. caninum* infections are known causes of reproductive failure (i.e. abortion, stillbirths and perinatal mortality) in different domestic and wild ruminant species [9, 10, 13, 14, 17, 65] but only few studies were performed to elucidate their significance in SAC. In Peru, *N. caninum* DNA and associated histological lesions were detected in the brain from three out of 15 aborted SAC foetuses (i.e. 2/6 alpaca and 1/9 llama foetuses) from the Puno region [19]. Additionally, *N. caninum* infection was also detected by PCR or immunohistochemistry in further 14/50 aborted SAC foetuses (i.e. 10/32 alpacas and 4/18 llamas) from the Central and South Andean regions of the Peruvian Highlands, of which eight foetuses also showed compatible lesions [18]. On the other hand, the role of *T. gondii* as cause of abortion in SAC has been subject of debate [57]. Two naïve llamas infected either experimentally or naturally with *T. gondii* during pregnancy remained clinically normal and delivered healthy, seronegative crias, suggesting that no transplacental transmission of the parasite occurred [67]. Later, *T. gondii* DNA was not detected in any of 15 [19] or 50 aborted foetuses from alpacas and llamas from Peru (while *N. caninum* was detected in some of the abortions), providing presumptive evidence that *T. gondii* is less important as a cause of abortion in SAC than *N. caninum* [18]. However, in the USA, toxoplasmosis was implicated as a cause of abortion in llamas, and antibodies against *T. gondii* were detected in foetal fluids from aborted foetuses [68]. Moreover, in recent years, *T. gondii* was confirmed as the cause of abortion in a near full-term alpaca foetus [20]. Nevertheless, the real prevalence of *N. caninum* and *T. gondii* associated abortions in SAC might be often underestimated, as aborted foetuses from these animal species seem to be only rarely submitted to the laboratory for further diagnosis [24]. In our study, only one out of 11 farms which experienced abortions in SAC reported having sent abortion material for etiological investigation. Further studies to estimate the involvement of these parasites as cause of abortion in SAC in Switzerland are on-going.

## Conclusions

One commercial ELISA kit for detection of antibodies against *T. gondii* and two kits for detection of *N. caninum* antibodies were assessed for their use in SAC and their cut-offs were optimized, showing almost perfect agreement with immunoblot results. One further commercial kit for the detection of antibodies against *N. caninum* in ruminants proved not to be useful for testing SAC. This nationwide cross-sectional study demonstrates, to our knowledge for the first time, the presence of antibodies against *T. gondii* and *N. caninum* in the Swiss SAC population and shed some light on the current epidemiological situation of these parasites, highlighting the occurrence of a low seroprevalence of *N. caninum* infection and a very high seroprevalence of *T. gondii* infection in llamas and alpacas in Switzerland, suggesting that SAC meat might represent an additional infection source for humans.

## Supplementary information

**Additional file 1: Table S1.** Relation of putative risk factors with the serological status for *N. caninum* infection in South American camelids from 41 Swiss farms, considered at the farm level.

## Data Availability

The datasets used and/or analysed during the current study are available from the corresponding author on reasonable request.

## Abbreviations

SAC: South American camelids
ELISA: enzyme-linked immunosorbent assay
TOXO-MS: ID Screen® Toxoplasmosis Indirect ELISA kit ID.vet
NCS-MS: ID Screen® Neospora caninum Indirect Multi-species ELISA kit ID.vet
NCC: ID Screen® Neospora caninum Competition ELISA kit ID.vet
NCS: ID Screen® Neospora caninum Indirect ELISA kit ID.vet
iv: intravenously
dpi: days post-inoculation
S/P%: sample-to-positive ratio
S/N%: competition percentage
OD: optical density
kDa: kilodaltons
v: volume
PBS: phosphate-buffered saline solution
ROC: receiver operating characteristics
AUC: area under the ROC curve
H0: null hypothesis
AIC: Akaike information criterion
IFAT: indirect fluorescent antibody test
FITC: fluorescein isothiocyanate conjugate
IHA: indirect hemagglutination
MAT: modified agglutination test
SE: standard error
CI: confidence interval
