# Supplementary material for: Toxoplasma gondii and Neospora caninum infections in South American camelids in Switzerland and assessment of serological tests for diagnosis
Source: Parasit Vectors. 2020 May 14;13:256. doi: 10.1186/s13071-020-04128-9 (PMC7227098; doi:10.1186/s13071-020-04128-9)
Supplement: Supplementary file 1 — Additional file 1: Table S1. Relation of putative risk factors with the serological status for N. caninum infection in South American camelids from 41 Swiss farms, considered at the farm level. [file 13071_2020_4128_MOESM1_ESM.docx]

**Additional file 1: Table S1**

Relation of putative risk factors with the serological status for *N. caninum* infection in South American camelids from 41 Swiss farms, considered at the farm level.

| Putative risk factor | Serological status for *N. caninum* at the farm level | |  |
| --- | --- | --- | --- |
|  | negative | positive | Total No. of farms |
| Origin of SAC bred in the farm |  |  |  |
| - only Swiss | 30 | 6 | 36 |
| - Swiss and imported (i.e. Peru, Chile, USA, Australia, New Zealand, Germany) | 5 | 0 | 5 |
| - Total farms | 35 | 6 | 41 |
| Presence of own cats in the farm during the last two years |  |  |  |
| - Yes | 30 | 5 | 35 |
| - No | 4 | 1 | 5 |
| - No answer | 1 | 0 | 1 |
| - Total farms | 35 | 6 | 41 |
| Number of cats in the farm during the last two years |  |  |  |
| - 1-4 cats | 21 | 4 | 25 |
| - 5-10 cats | 7 | 1 | 8 |
| - >10 cats | 2 | 0 | 2 |
| - Total farms | 30 | 5 | 35 |
| Access of own cats to stable and pastures |  |  |  |
| - Yes | 29 | 5 | 34 |
| - No | 1 | 0 | 1 |
| - Total farms | 30 | 5 | 35 |
| Access foreign cats to the farm: |  |  |  |
| - Yes | 28 | 5 | 33 |
| - No | 2 | 0 | 2 |
| - Total farms | 30 | 5 | 35 |
| Kittens (≤6 months) were present in the farm in the last two years |  |  |  |
| - Yes | 7 | 1 | 8 |
| - No | 23 | 4 | 27 |
| - Total farms | 30 | 5 | 35 |
| Presence of own dogs in the farm in the last two years |  |  |  |
| - Yes | 18 | 4 | 22 |
| - No | 17 | 2 | 19 |
| - Total farms | 35 | 6 | 41 |
| Access of own dogs to stable and pasture: |  |  |  |
| - Yes | 17 | 4 | 21 |
| - No | 1 | 0 | 1 |
| - Total farms | 18 | 4 | 22 |
| Access of foreign dogs to the farm |  |  |  |
| -Yes | 10 | 1 | 11 |
| - No | 9 | 3 | 12 |
| - No answer | 16 | 2 | 18 |
| - Total farms | 35 | 6 | 41 |
| Presence of puppies (≤6 months) in the farm in the last two years |  |  |  |
| - Yes | 2 | 0 | 2 |
| - No | 16 | 4 | 20 |
| - Total farms | 18 | 4 | 22 |
| Problems with rodents (mice or rats) in the farm |  |  |  |
| - Yes | 7 | 0 | 7 |
| - No | 26 | 6 | 32 |
| - No answer | 2 | 0 | 2 |
| - Total | 35 | 6 | 41 |
| Breeding of other animal species in the farm |  |  |  |
| - Yes | 29 | 5 | 34 |
| - No | 6 | 1 | 7 |
| - Total | 35 | 6 | 41 |
| Occurrence of abortions in SAC |  |  |  |
| - Yes | 11 | 0 | 11 |
| - No | 24 | 6 | 30 |
| - Total | 35 | 6 | 41 |
| Number of abortions |  |  |  |
| - 0 | 24 | 6 | 30 |
| - 1 | 7 | 0 | 7 |
| - 2 | 3 | 0 | 3 |
| - 5 | 1 | 0 | 1 |
| - Total farms | 35 | 6 | 41 |
| Sending abort material for investigation |  |  |  |
| - Yes | 1 | 0 | 1 |
| - No | 10 | 0 | 10 |
| - Total | 11 | 0 | 11 |
| Water supply |  |  |  |
| - Creek | 2 | 0 | 2 |
| - well | 7 | 0 | 7 |
| - Bucket drinker | 10 | 2 | 12 |
| - Tap water | 8 | 2 | 10 |
| - Tap water and creek | 1 | 0 | 1 |
| - Tap water and well | 1 | 0 | 1 |
| - Automatic drinker | 3 | 2 | 5 |
| - Combinations of the former | 3 | 0 | 3 |
| - Total farms | 35 | 6 | 41 |
| Type of feeding |  |  |  |
| - Pasture | 34 | 5 | 39 |
| - Fresh grass | 2 | 0 | 2 |
| - Hay | 35 | 6 | 41 |
| - Straw | 3 | 1 | 4 |
| - Grass silage | 6 | 0 | 6 |
| - Corn silage | 2 | 0 | 2 |
| - Concentrated feed | 13 | 3 | 16 |
| - Total farms | 35 | 6 | 41 |
| Silage storage (only farms feeding silage) |  |  |  |
| - Silo bales | 5 | 0 | 5 |
| - Horizontal silo | 2 | 0 | 2 |
| - Other | 1 | 0 | 1 |
